# The Kinetic Study of the Influence of Common Modifiers on the Curing Process of Epoxy Vitrimers

**DOI:** 10.3390/polym16030392

**Published:** 2024-01-31

**Authors:** Roman Korotkov, Vyacheslav Shutov, Alexey Orlov, Natalia Bornosuz, Daria Kulemza, Denis Onuchin, Anna Shcherbina, Irina Gorbunova, Igor Sirotin

**Affiliations:** 1 Faculty of Petrochemistry and Polymer Materials, Mendeleev University of Chemical Technology, Miusskaya Sq. 9, 125047 Moscow, Russia; roman.korotkov@pccl.at (R.K.); sherbina.a.a@muctr.ru (A.S.); gorbunova.i.i@muctr.ru (I.G.); 2 Polymer Competence Center Leoben GmbH, 8700 Leoben, Austria

**Keywords:** vitrimers, curing kinetics, transesterification, curing catalyst, epoxy

## Abstract

An analysis of the influence of common modifiers on the kinetics of the curing process of epoxy-anhydride vitrimers was carried out. As common modifiers to enhance the “vitrimeric” nature of the material, zinc acetylacetonate as a transesterification catalyst and glycerol as a modifier of hydroxyl group content were chosen. The curing process of all obtained compositions was studied by differential scanning calorimetry (DSC) followed by the application of the isoconversional approach. It was shown that additives significantly affect the curing process. The resulting cured polymers were shown to be chemically recyclable by dissolution in the mixture of ethylene glycol and N-methylpirrolidone in a volume ratio of nine to one. The introduction of both zinc acethylacetonate and glycerol to the neat formulation led to a decrease in the dissolution time by 85.7% (from 35 h for the neat epoxy-anhydride formulation to 5 h for the modified formulation). In order to show the opportunity of the secondary use of recyclates, the mixtures based on the basic composition containing 10 wt. % of secondary polymers were also studied. The introduction of a recycled material to neat composition led to the same curing behavior as glycerol-containing systems.

## 1. Introduction

Nowadays, polymer-matrix composites (PMCs) are widely used in different areas of industry, such as aerospace, wind energy, automotive, construction, and others. The application of PMCs is widely spread due to their mechanical characteristics, resistance to corrosion and chemical interaction, dimensional stability during operation, and relatively low weight [1,2,3]. Among the most common thermosetting resins for PMC usage, epoxy resin occupies an important place. While it appeared on the market in the late 1940s, it still plays a key role in various industrial and commercial purposes. The processing of epoxy resins is characterized by universal technological properties, a sufficiently low shrinkage, and a curing without volatiles. Epoxy plastics have good chemical and corrosion resistance and high adhesive and thermomechanical properties [4,5,6].

Regarding PMCs in terms of sustainable development, we come across an old issue of composite recycling. The roots of this problem are fundamental and stem from a distinctive property of conventional cured thermosetting matrices, which is a complete and irreversible loss of fluidity, which does not allow them to be secondarily processed like thermoplastics. Therefore, nowadays, researchers all over the world are prompted by ecological challenges to address this problem. The main directions of the development are the repurposing of end-of-life composites, improving technologies of fiber recovery [7], and, of course, the invention of a new matrix to be used in composites for easier recycling. In addition, there are a lot of challenges correlated to the closing of the loop on composite materials that cover ecology, marketing, business, philosophy, etc., which are closely interrelated. This is also associated with a problem of collection and sorting of PMC wastes. There are three main approaches in the recycling of composites: mechanical, thermal, and chemical [8]. In order to implement mechanical recycling, composites are ground and used as an additive filler for other materials. The chemical method mainly involves the dissolution of a polymer matrix and the recovery of reinforcing fibers and some of the matrix components. There is also highly efficient recycling technology utilizing supercritical fluids. However, the most popular but not resilient method commercialized is pyrolysis [9,10]. At present, the economic feasibility of composite recycling lies in the reclamation of carbon fibers. Methods enabling the recovery of a matrix and its components are not used due to economic reasons as raw materials are mostly cheaper than secondary ones. In fact, the above-mentioned challenge of chemical recycling is the problem that our research is struggling to address with the help of a matrix that is based on smart materials called vitrimers. These materials are capable of undergoing plastic deformation under certain conditions and are self-healing, which enables a secondary molding of composite wastes based on vitrimers, similar to thermoplastics. In some cases, a complete dissolution of a matrix is possible, which enables the recovery of both a fiber and a resin without large economic costs.

Thus, we give credit to vitrimers as a material that could become crucial in sustainable and resilient composite production. The feature of vitrimer nature is dynamic covalent bonds that are known in the literature as covalent adaptable networks (CANs) [11,12,13]. At the operating temperature, vitrimer plastics behave like conventional thermosets with good thermal and mechanical properties. When heated up to the topology freezing point (T_v_), the exchange reaction quickly proceeds and, under load, it causes the rearrangement of topology and rapid stress relaxation in the material, as a result of which some epoxy vitrimers can “flow” like a viscoelastic liquid [14]. These unique properties make them similar to thermoplastic materials [12,15,16] and expand applications of thermosetting PMCs.

Recently, many different types of CANs were described. All the described mechanisms can be divided into two classes: associative and dissociative CANs [17]. Dissociative CANs can be rearranged through reversible reactions in which the chemical bond is broken in one place and recovered in another [18]. However, such an exchange reaction leads to a decrease in the degree of crosslinking when chemical bonds break and, as a consequence, to a dramatic drop in viscosity, as a result of which the network may lose its dimensional integrity and resistance to solvents [15,19]. Associative CANs do not depolymerize and remain integrated since the exchange reactions proceed through the intermediate formation stage. The degree of crosslinking of the matrix in this case remains constant [20]. Currently, more than 30 types of associative CAN mechanisms are known [21], including transesterification, disulfide exchange [22,23], olefin metathesis [24,25], transalkylation exchange [26,27], exchange of boronic acid esters [28,29], trans carbamoylation of aromatic polyurethanes [30], polyhydroxyurethanes [31] or poly(oxime-urethane) [32], tritiocarbonate exchange [33], transamination of vinylological amides or urethanes [34], imine exchange in polyimines [35,36,37,38], Michael’s thiol reaction [39], alkoxyamine moieties [40], and carbonate exchange [41].

**Figure 1:**
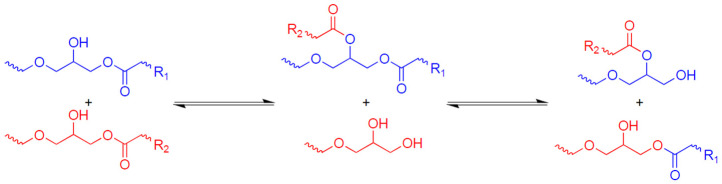
The transesterification mechanism in vitrimers.

The first paper dedicated to vitrimers [42] was an investigation of materials based on the transesterification mechanism presented in Figure 1. Transesterification is a classical reaction of organic chemistry, which is widely known as an equilibrium reaction in which an ester and an alcohol are converted into another ester and another alcohol through the exchange of alkoxy substituents.

The main issue of transesterification-based vitrimers is the insufficient reactivity needed for good re-processable properties. Typical ways of properties regulation of these vitrimers are the use of catalysts [43], the increase in the concentration of hydroxyl groups [44,45,46], and a decrease in the crosslink density [47,48]. Many catalysis mechanisms are known for the transesterification reaction: acidic, basic, catalysis by amines, organometallic compounds, and enzymes [49]. Thus, there are plenty of compounds that could catalyze transesterification reactions in vitrimers, for instance, zinc salts [43,44,47,50,51,52], triphenylposphine [43], triazobicyclodecene [43], and tertiary amines [53]. An increase in the hydroxyl group content could be achieved by different approaches: the addition of polyhydric alcohol to epoxy-anhydride resin [44], the use of oligomeric epoxy resins enriched with hydroxyl groups [45], and the use of carboxylic acid as a hardener [54].

In this study, we reported the investigation on epoxy-anhydride vitrimers based on transesterification. This study reveals the influence of glycerol and zinc acetylacetonate addition to DER-330 epoxy resin with i-MTHPA anhydride hardener with 4-methylimidazole as the curing catalyst on the kinetics of curing, recycling, and technological properties of resin compositions. The influence of selected modifiers on the curing and performance of epoxy-anhydride resins is still not well-investigated. The studied base composition is widely used for PMC production, and the use of such modifiers can increase the sustainability of the formulation by the improvement of its recyclability that was studied by chemical dissolution of obtained cured formulations in the mixture of ethylene glycol and N-methylpirrolidone via transesterification reaction. For the investigation, zinc acetylacetonate, as one of the most published catalysts for the transesterification reaction, and glycerol, as a modifier that significantly increases the concentration of hydroxyl groups and hence shifts the equilibrium of the transesterifications were chosen. As was shown in [44], the addition of glycerol led to a decrease in the glass transition temperature of epoxy vitrimers. Thus, to eliminate this effect, glycerol was added in the minimal effective amount based on [44]. The zinc acetylacetonate was added in an amount of 5 % mol. based on [43]. The curing process of all obtained compositions was studied by differential scanning calorimetry (DSC) at different heating rates followed by application of the isoconversional and model-based approaches. Besides that, in order to investigate the possibilities of epoxy recirculate use as a modifier, the formulation containing 10 wt. % of chemically recycled epoxy resin was prepared and studied for comparison.

## 2. Materials and Methods

### 2.1. Raw Materials

Epoxy resin DER-330 with epoxy equivalent 180 EE was supplied by Dow Chemical Company, Midland, MI, USA. Glycerol (99%) was purchased from the chemical company BASF, Ludwigshafen, Germany; iso-MTHPA hardener (i-MTHPA, 99%) was supplied by Chimex Ltd., St. Petersburg, Russia; and 4-methylimidazole (4-MIm, 99%) was purchased from Sigma Aldrich, Burlington, MA, USA. Anhydrous zinc acetylacetonate (Zn(AcAc)_2_) was synthesized according to methods known in the literature [55]. Ethyleneglycole (EG, 99.9%), N-methylpirrolidone (NMP, 99%), acetone, dichloromethane, toluene, ethanol, chloroform, dioxane, and tetrahydrofurane (THF) were purchased from Rushim, Moscow, Russia. All chemicals were used as received without further purification.

### 2.2. Preparation of Resin Compositions

The initial matrix was chosen to be an epoxy-anhydride type with imidazole as a catalyst. The amount of i-MTHPA was added in accordance with TDS as 85 g per 100 g of DER-330. In addition, 4-MIm was introduced to the mixture in the amount of 1 wt. %, which is 4.5 mol % of the amount of anhydride. In order to determine the influence of additives on the recycling process, technological and mechanical characteristics the compositions presented in Table 1 were prepared. The equivalent mole ratio for EP (DER-330:i-MTHPA:4-MIm) was 1:2:0.09; for EP-G (DER-330:i-MTHPA:4-MIm:glycerol), it was 1:1.05:0.046:0.326.

Compositions EP and EP-G were prepared by the following method. A calculated amount of DER-330, 4-MIm, and glycerol was placed in a single-necked round-bottomed flask. The mixture was stirred at a temperature of 50 °C until a homogeneous mixture was obtained. After stirring, the mixture was cooled to room temperature and the required amount of i-MTHPA was added, and the resulting mixture was stirred for 10 min. Subsequent degassing of the systems was performed at 30 °C on a vacuum rotary evaporator at a residual pressure of 1.0 kPa for 20 min.

Compositions EP-Z and EP-GZ were prepared by the following method. A calculated amount of DER-330 and Zn(AcAc)_2_ was placed in a single-necked round-bottomed flask and stirred at a temperature of 85 °C until a transparent homogeneous liquid was obtained. Next, the mixture was cooled to 50 °C, and the required amount of 4-MIm was added. The resulting composition was stirred until the transparent homogeneous liquid was obtained. The obtained mixture was cooled to room temperature, and the required amount of glycerol and i-MTHFA was added. The resulting mixture was stirred for 10 min. Subsequent degassing of the systems was performed at 30 °C on a vacuum rotary evaporator at a residual pressure of 1.0 kPa for 20 min.

Obtained compositions were cast to plates with the dimensions 170 mm × 170 mm × 2 mm and cured in a closed mold according to the following regime: 100 °C—1 h, 120 °C—3 h, 150 °C—7 h.

In order to conduct further experiments and tests with the cured compositions, specimens of the required sizes were cut from the plates by a CNC milling machine (Jinan Wattsan CNC Technology Co., Ltd., Jinan, China).

The compositions containing recycled resin were prepared by the following method. Calculated amounts of DER-330, 4-MIm, and recycled resin (10 wt. %) were transferred in a single-necked round-bottomed flask and stirred at a temperature of 50 °C until a homogeneous mixture was formed. After stirring, the mixture was cooled to room temperature and the required amount of i-MTHPA was added. The resulting mixture was stirred for 10 min followed by a degassing of the system, which was performed at 30 °C on a vacuum rotary evaporator at a residual pressure of 1.0 kPa for 20 min.

### 2.3. Dissolution of the Cured Compositions and Resin Recovery from the Solution

The recycling of cured epoxy–vitrimer compositions was carried out as follows: in a laboratory plastic crusher, vitrimer plastic was ground to a particle size range of 0.5–2.0 mm; 8 g of the resulting powder and 200 mL of a mixture of ethylene glycol and N-methylpyrrolidone in a mass ratio of 9:1 were transferred to a single-necked round-bottomed flask with a volume of 500 mL. The resulting suspension was boiled until the solid particles were completely dissolved. After dissolution and cooling to room temperature, the resulting solution was evaporated and then precipitated into distilled water. The precipitate was extracted into dichloromethane, after which the solution was dried from residual water over anhydrous sodium sulfate and evaporated.

### 2.4. Measurements

A differential scanning calorimeter DSC 214 Polyma (Netzsch, Selb, Germany) was employed for monitoring thermal effects. In order to study a process of curing, several heating rates 5, 10, and 20 K/min were used, and the data was analyzed according to ISO 11357-5:2013 [56]. Glass transition temperatures of cured samples were determined in accordance with ISO 11357-2:2020 [57] at a heating rate of 10 K/min. All tests were performed in a nitrogen atmosphere at a flow rate of 40 mL/min with sensitivity and temperature calibrations applied. An aluminum Concavus^®^ pan with a pierced lid with a pinhole size of approximately 0.7 mm was used. The weight of samples ranged from 5 to 10 mg. For data processing, Proteus Thermal Analysis version 8.0.2 software (Netzsch, Selb, Germany) was used. As a result of every measurement, several key points were defined: onset temperature (T_onset_), defined as the intersection of two tangent lines to the DSC curve at the point before peak started and the point with maximum peak slope; peak temperature (T_peak_), defined as peak maximum; peak end temperature (T_end_), defined as an intersection of two tangent lines to the DSC curve at the point with maximum peak slope and the point after peak ended; reaction enthalpy (ΔH), defined as peak area; and T_g_ middle, defined as inflection point of DSC curve.

Rheological experiments were carried out on the Anton Paar MCR 302 modular rheometer (Graz, Austria) in the oscillatory mode with a frequency of 1 Hz, a shear strain of 1%, and a gap of 1 mm using plate–plate geometry with a heating rate of 5 K/min.

Glass transition temperatures and temperature dependencies of storage (E′) and loss (E″) moduli were determined following the procedure specified in ASTM D7028-07 [58], using DMA GABO Eplexor 25N (Netzsch, Selb, Germany) dynamic mechanical analyzer. Measurements were performed in three-point bending mode within the temperature range of 30–200 °C, at a heating rate of 2 °C/min, static strain of 10%, oscillation frequency of 1 Hz, and strain amplitude of 8%.

Stress-relaxation tests were performed using Netzsch DMA GABO Eplexor 25N at different temperatures. A sample of cured resin was fixed in tensile clamps. After heating, the load of 20 N was applied to the sample, and the relaxation curve was obtained.

Tensile tests of cured resin were performed using the universal tensile machine 50ST (Tinius Olsen, Salfords, Great Britain) in accordance with ISO 527-2:2012 [59] at a crosshead speed of 1 mm/min.

Statistical analysis of mechanical test results was conducted in accordance with ISO 2602-1980 procedure [60] for two-sided confidence intervals with a confidence level of 0.95.

### 2.5. Kinetic Computations

The recommendations of the ICTAC Kinetic Committee were followed to evaluate the dependence of the activation energy on the extent of conversion as well as the implementation of the model approach [61]. The values of the extent of conversion, α, were determined as the partial areas $Q(t)/Q_{total}$ of the DSC peaks associated with the curing process, where Q(t) is the current heat change up to a certain point in time, t, and $Q_{total}$ is the total possible heat of the process.

The dependence of the activation energy, $E_{\alpha }$, on the extent of conversion, α, was evaluated using the flexible integral isoconversional method of Vyazovkin [62]. This method eliminates the systematic error in the calculation of the dependence of the activation energy, which is introduced due to the fact that the activation energy is not constant over the whole interval of integration and quite commonly varies with α. Thus, the procedure for estimating the dependence of $E_{\alpha }$ on α lies in finding the minimum of the following function:

(1) $$\Psi \left(E_{\alpha }\right)=\sum_{i=1}^{n}\sum_{j\neq i}^{n}\frac{J\left(E_{\alpha },T_{\alpha ,i}\right)\beta_{j}}{J\left(E_{\alpha },T_{\alpha ,j}\right)\beta_{i}}$$

where

(2) $$J(E_{\alpha },T_{\alpha })=\int_{T_{\alpha -∆\alpha }}^{T_{\alpha }}exp\left(-\frac{E_{\alpha }}{RT}\right)dT$$

is the temperature integral [63]. Integration is flexible in this case and performed over small intervals of either temperature, ∆T, or time, ∆*t*, which correspond to small intervals of ∆α. The constancy of $E_{\alpha }$ is now assumed in a very narrow range of integration, Δ*α*, which was taken as 0.025. Minimization of (1) is repeated for each value of *α* to obtain a dependence of $E_{\alpha }$ on α.

The minimization procedure was performed in the MATLAB^®^ R2019b workspace using the Nelder–Mead simplex direct search method (fminsearch function). Numerical calculation of the temperature integral (2) was carried out using global adaptive quadrature and default error tolerances: absolute error tolerance had the value of 1 × 10^−10^ and relative error tolerance had the value of 1 × 10^−6^.

The model-based analysis presented in this paper was performed using NETZSCH Thermokinetics 3.1 software. The reaction rate in the condensed phase can be represented by the following equation [61]:

(3) $$\frac{d\alpha }{dt}=k\left(T\right)f\left(\alpha \right)$$

where α is the extent of conversion of the reactant to the products, *t* is the time, T is the absolute temperature, and $k\left(T\right)$ is the Arrhenius temperature-dependent rate constant. Here, $f\left(\alpha \right)$ is a reaction model. For the model-based analysis extended Prout–Tompkins model,

(4) $$f\left(\alpha \right)={\left(1-\alpha \right)}^{n}\alpha^{m}$$

was initially chosen for its ability to describe the autocatalytic kinetics of processes.

## 3. Results and Discussion

### 3.1. Study of Curing Process

**Figure 2:**
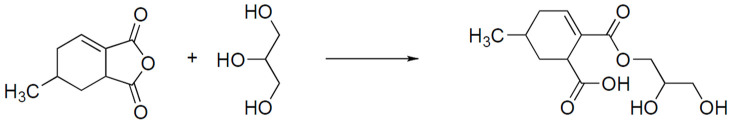
The proposed mechanism of reaction between glycerol and isomers of methyltetrahydrophtalic anhydride.

Glycerol can react with anhydrides with the opening of the anhydride cycle, resulting in the formation of an acid group, thereby catalyzing the curing process. The proposed mechanism of the reaction of glycerol and anhydride is shown in Figure 2. Since the addition of glycerol significantly reduces the glass transition temperature of the cured matrix by reducing the degree of crosslinking and introducing a flexible aliphatic fragment, the glycerol content is limited to 10 mass parts per 100 mass parts of DER-330 resin, which corresponds to 0.2 mol glycerol per 1 mol of the epoxy group. According to [44], the introduction of 0.25 mol of glycerol per 1 mol of the epoxy group already has a significant effect on the relaxation time of the epoxy vitrimer. The introduction of zinc salts also has a significant effect on the relaxation time of the epoxy vitrimer. According to [43], for compositions containing 5 and 10 molar % zinc salt, there is practically no difference in relaxation times. The concentration of zinc acetylacetonate in the compositions EP-Z and EP-GZ is 6.73 mass parts per 100 parts of the epoxy resin, which corresponds to 0.05 mol per 1 mol of ester groups obtained as a result of the complete conversion of iso-MTHPA.

**Figure 3:**
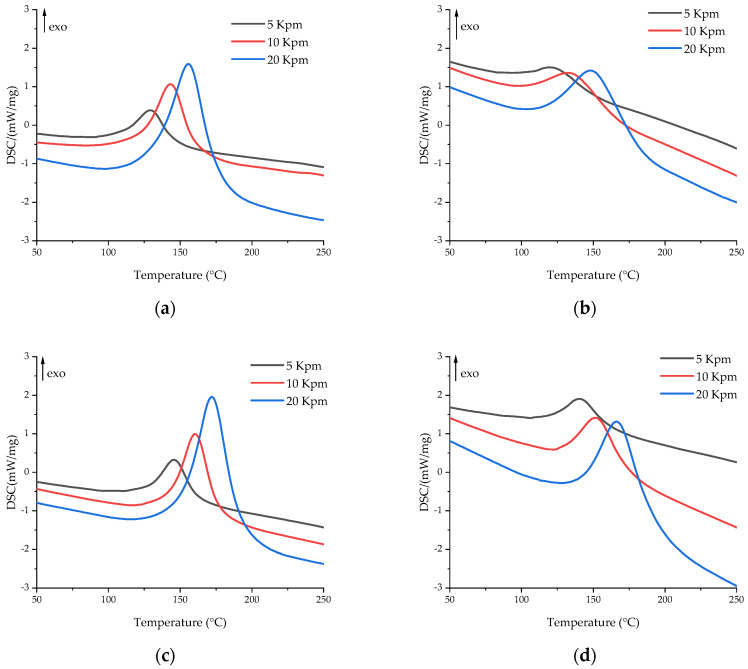
DSC curves of uncured compositions at heating rates of 5 (black), 10 (red), and 20 (blue) K/min for (**a**) EP, (**b**) EP-G, (**c**) EP-Z, and (**d**) EP-GZ.

The effects of glycerol and zinc acetylacetonate on the curing process of the epoxy anhydride resin were studied by DSC and rheology methods. The DSC curves are shown in Figure 3. The key temperature characteristics of the DSC scans are presented in Table 2. According to the DSC data, additives have a significant impact on the curing process of compositions.

Glycerol accelerates the polymerization process due to the participation of its hydroxyl groups in the anhydride ring-opening reaction, which leads to the formation of an acid group, which also exhibits a significant catalytic effect [44]. While the amount of 4-methylimidazole to anhydride is 4.5 mol. %, the amount of hydroxyl groups in glycerol to anhydride is 31 mol. %, which leads to the quick formation of highly reactive acid groups at elevated temperatures, which in turn results in a simultaneous curing of epoxy resin. Therefore, in Figure 3 and Table 2, a significant shift of curing to the lower temperature region is observed. Moreover, the introduction of glycerol led to a decrease in reaction enthalpy, which also indicated a ring-opening reaction between –OH groups and anhydrides.

The addition of zinc acetylacetonate, on the contrary, significantly slows down the polymerization process. This effect may be associated with the formation of zinc complexes with a lone electron pair in carboxyl groups, hydroxyl groups, and imidazole, which impedes the curing process due to the electron-accepting nature of Zn^2+^ [64,65,66]. The concentration of Zn^2+^ of 5 mol. % to the anhydride group would not allow a significant change in the equilibrations of curing reactions by complexation of the carboxyl and hydroxyl groups and hence the curing rate of the resin composition. We assume that the increase in the onset temperature of the polymerization process could be caused by the formation of Zn^2+^–imidazole complexes. The imidazole complexes of 3d-metals show reduced catalytic activity during the curing of the epoxy resin [67].

The use of glycerol and zinc acetylacetonate together slows down the polymerization process, but not as much as individual acetylacetonate, due to the excess of glycerol compared to the amount of zinc acetylacetonate, which is added in the amount of 5 mol. % to the anhydride group. Thus, in comparison with EP-Z, the polymerization of EP-GZ proceeds in a lower temperature region, but compared to EP-G and EP, the curing peak shifts to higher temperatures due to the hindrance of a zinc complex formation.

**Figure 4:**
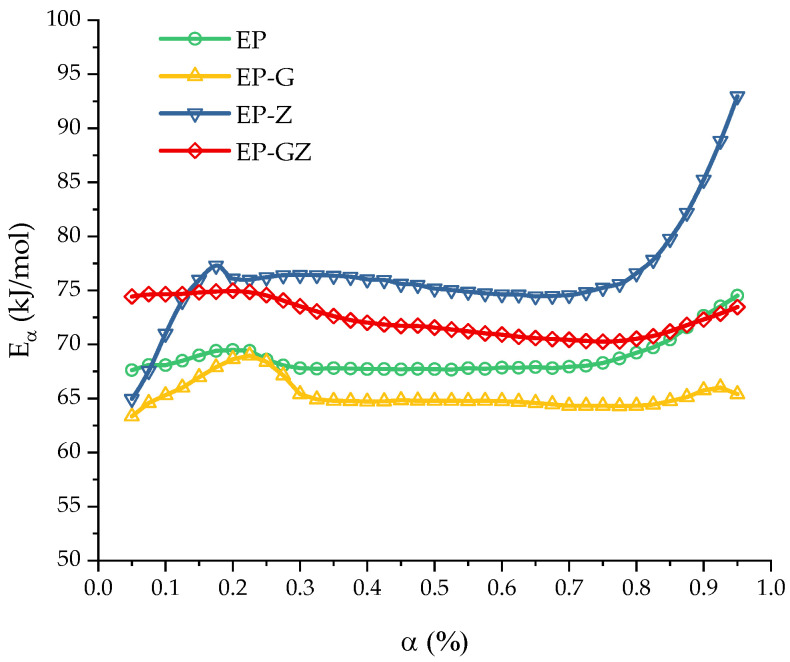
Activation energy $E_{\alpha }$ of the curing as a function of the extent of conversion α computed for all compositions.

For a more detailed and quantitative investigation of the polymerization process, a study of the kinetics of the curing process was conducted. In Figure 4, the dependences of the isoconversional activation energy for all compositions are presented.

The curing process of the EP, EP-G, and EP-GZ compositions demonstrates a slight variation of the activation energy with the extent of conversion α in the range of 0.05–0.95. The constancy of the dependence of the activation energy in a certain region indicates that the curing process here is probably controlled by a single reaction step.

The curing of the EP system was characterized by an average value of $E_{\alpha }=68.8\;\pm \;0.28\;kJ/mol$. The introduction of glycerol into the system accelerates the curing process, respectively, reducing the activation energy of curing to $E_{\alpha }=65.32\;\pm \;0.22\;kJ/mol$. This effect was associated with the opening of the anhydride ring by the hydroxyl groups of glycerol. The additional amount of the free carboxyl groups formed (in addition to those formed by introducing imidazole into the system) accelerates the epoxide ring-opening reaction, in comparison with the course of the reaction in the initial anhydride–imidazole system.

The curing process of EP-Z is characterized by the constancy of the effective activation energy only in the region of 0.15–0.70, with an average value of $E_{\alpha }=75.6\;\pm \;0.17\;kJ/mol$. The presence of glycerol in the reaction medium (EP-GZ) accelerates this process compared to EP-Z; thus, the activation energy of this process is lower than the activation energy of the curing process of EP-Z and is ~72.4±0.27kJ/mol. Overall, it is noteworthy that the calculated activation energy dependencies correlate well with the suggestions made above regarding the influence of additives on the curing processes. The mean activation energies are presented in Table 3.

The curing reaction of the vitrimers was autocatalytic for all the compositions. This is proved by the fact that the obtained kinetic data were successfully described by autocatalytic models. In the present work, the following autocatalytic models were initially used:

(5) $$\frac{d\alpha }{dt}=Ae^{-\frac{E}{RT}}{\left(1-\alpha \right)}^{n}\alpha^{m}$$

(6) $$\frac{d\alpha }{dt}=Ae^{-\frac{E}{RT}}{\left(1-\alpha \right)}^{n}\left(1+B\alpha \right)$$

**Figure 5:**
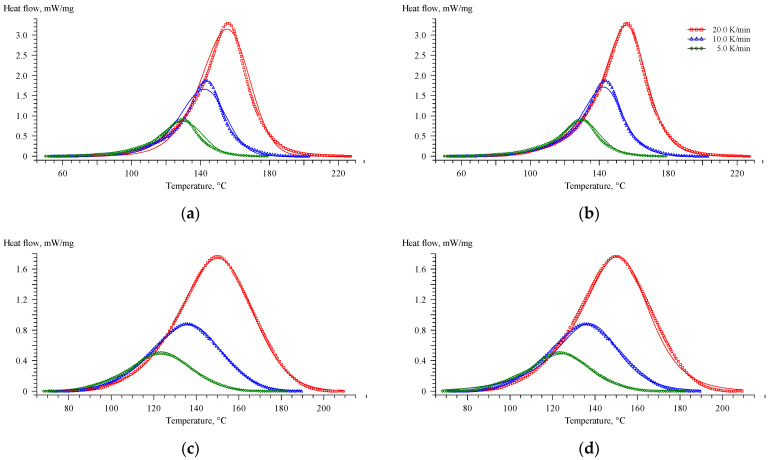
Comparison of the experimental DSC curves (symbols) with those estimated (solid lines) using the Bna (**a**,**c**) and Cn (**b**,**d**) models for EP (**a**,**b**) and EP-G (**c**,**d**) systems.

The kinetic function f(α) in the Equation (5) is the extended Prout–Tompkins or so-called Bna reaction model. The kinetic function ${\left(1-\alpha \right)}^{n}(1+B\alpha )$ in Equation (6) is the so-called Cn reaction model. The results of fitting using the Bna and Cn models for the EP and EP-G systems are presented below (Figure 5).

As can be seen, the Cn model describes the curing process for the EP system well, while the kinetics of the process for EP-G is well described by the Bna model. However, it is much more convenient to compare the obtained kinetic parameters by using a uniform kinetic model to describe the curing process of all systems.

The Cn model (Equation (6)) is a special case of the general Kamal–Sourour model (Equation (7)),

(7) $$\frac{d\alpha }{dt}=A_{1}e^{-\frac{E_{1}}{RT}}\underset{Fn}{\underbrace{{\left(1-\alpha \right)}^{n}}}+A_{2}e^{-\frac{E_{2}}{RT}}\underset{Bna}{\underbrace{{\left(1-\alpha \right)}^{n}\alpha^{m}}}$$

which is a combination of n-order (*Fn*) and the extended Prout–Tompkins autocatalytic (*Bna*) kinetic equations.

With $B=A_{2}/A_{1}$, $E=E_{1}=E_{2}$, and m=1, the Kamal–Sourour model becomes the Cn model (6), which is written in the following form (Equation (8)):

(8) $$\frac{d\alpha }{dt}=Ae^{-\frac{E}{RT}}{\left(1-\alpha \right)}^{n}+BAe^{-\frac{E}{RT}}{\left(1-\alpha \right)}^{n}\alpha$$

Thus, to analyze the curing process of all systems, Equation (9),

(9) $$\frac{d\alpha }{dt}=A_{1}e^{-\frac{E}{RT}}{\left(1-\alpha \right)}^{n}+A_{2}e^{-\frac{E}{RT}}{\left(1-\alpha \right)}^{n}\alpha^{m}$$

which includes the characteristics of models (5) and (6), was chosen,

which is in fact the Cnm model:

(10) $$\frac{d\alpha }{dt}=Ae^{-\frac{E}{RT}}{\left(1-\alpha \right)}^{n}(1+B\alpha^{m})$$

**Figure 6:**
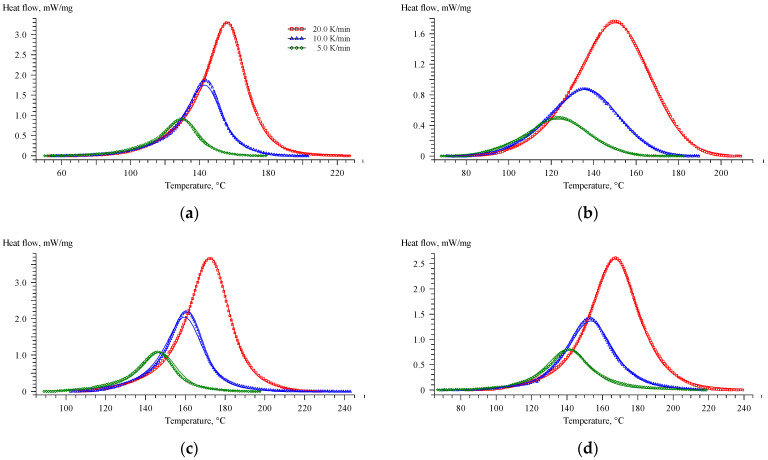
Comparison of the experimental DSC curves (symbols) with those estimated (solid lines) using the Cnm model for EP (**a**), EP-G (**b**), EP-Z (**c**), and EP-GZ (**d**) systems.

The results of fitting using the Cnm model are presented in Figure 6 and Table 4. Here, $A_{1}$ is the pre-exponential factor for the non-catalyzed reaction ${\left(1-\alpha \right)}^{n}$, and $A_{2}$ is the pre-exponential factor for the catalyzed path ${\left(1-\alpha \right)}^{n}\alpha^{m}$. It should be noted that the chosen model (Equation (10)) has an important advantage over the autocatalytic model (5). The constant B in the model (Equation (10)), which is the ratio of the pre-exponential factors of two competing reactions with assumed equal activation energies, shows how many times the rate constant of the autocatalytic reaction exceeds the rate constant of the *n*-th order reaction.

In the case of EP, EP-Z, and EP-GZ systems, the factor B is close to 10, which means that the contribution of the autocatalytic reaction to the effective reaction rate constant is great and close to 90%. For the EP-G system, the pre-exponential factor $A_{1}\to 0$; therefore, such a reaction can be considered as quasi one-step process. From the data obtained using the model (Equation (10)) follows that a decrease in the activation energy of the curing process was observed for systems containing glycerol—for system EP-G in relation to system EP and for system EP-GZ in relation to system EP-Z. As mentioned above, this is due to the fact that the hydroxyl groups of glycerol catalyze the curing reaction mainly due to anhydride cycle opening.

It is worth noting that the obtained values of the activation energy are in good agreement with those obtained using the isoconversional method.

**Figure 7:**
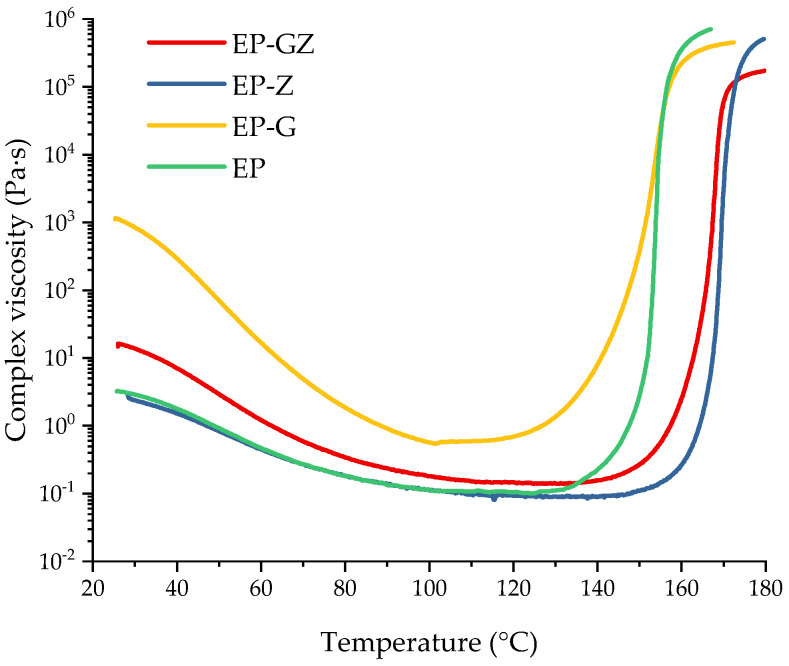
Complex viscosity dependences on the temperature of uncured compositions.

In addition, the curing process was investigated using the rheometry method. The temperature dependences of complex viscosity change are shown in Figure 7. In general, it can be seen that the tendency of viscosity changes during curing for different compositions correlates to the change in the extent of conversion calculated from DSC data, whereas the change in the initial viscosity is different.

The initial composition had the value of complex viscosity of 2.5 Pa·s at room temperature (RT). No reaction occurred at this stage. The introduction of glycerol significantly increased the viscosity value at RT, although this value for the glycerol was 1 Pa·s at RT. We suggest that this is associated with the formation of intermediates (Figure 2) as a result of the reaction between glycerol and i-MTHFA, which, with the development of the reaction, may have a branched structure. The introduction of zinc acetylacetonate into the composition, on the contrary, slightly affected the complex viscosity at RT, which indicated that no complex formation occurred due to the lack of functional groups. As for the EP-GZ composition, there are several reactions that proceed at RT. This is the interaction of glycerol and anhydride with the formation of carboxyl groups and the complexation of hydroxyl and carboxyl groups with zinc. EP-GZ demonstrates a lower viscosity at RT compared to EP-G, which can be explained by the participation of the hydroxyl groups of glycerol in the complex formation that prevents them from reacting with anhydride completely, resulting in the formation of a less branched structure of intermediate.

The gel points of the compositions at a heating rate of 5 °C/min were calculated from the viscosity data as the intersection of the abscissa line and the tangent to the viscosity curve at the point of maximum viscosity increase rate. The gel point of EP was at 154 °C. The gel point of the EP-G was slightly lower (at 153 °C) because of the acceleration of the curing process by the free hydroxyl groups of glycerol. Although it was previously shown that with the introduction of glycerol into the system the curing reaction is accelerated, the fact that the gelation point remained almost unchanged can be explained as follows. Apparently, with the introduction of glycerol, a more flexible network is formed, which in turn leads to an increase in the time interval between the onset of the reaction and the onset of loss of fluidity for the system with glycerin.

The gelation of EP-Z and EP-GZ occurred only at 170 °C and 168 °C, respectively, which could be caused by the assumed Zn^2+^–imidazole complex formation, decreasing the equilibration of an imidazole-induced anhydride ring-opening reaction and hence shifting the onset of polymerization. This is consistent with the DSC data of the curing process of the compositions.

### 3.2. Properties of Cured Resins

**Figure 8:**
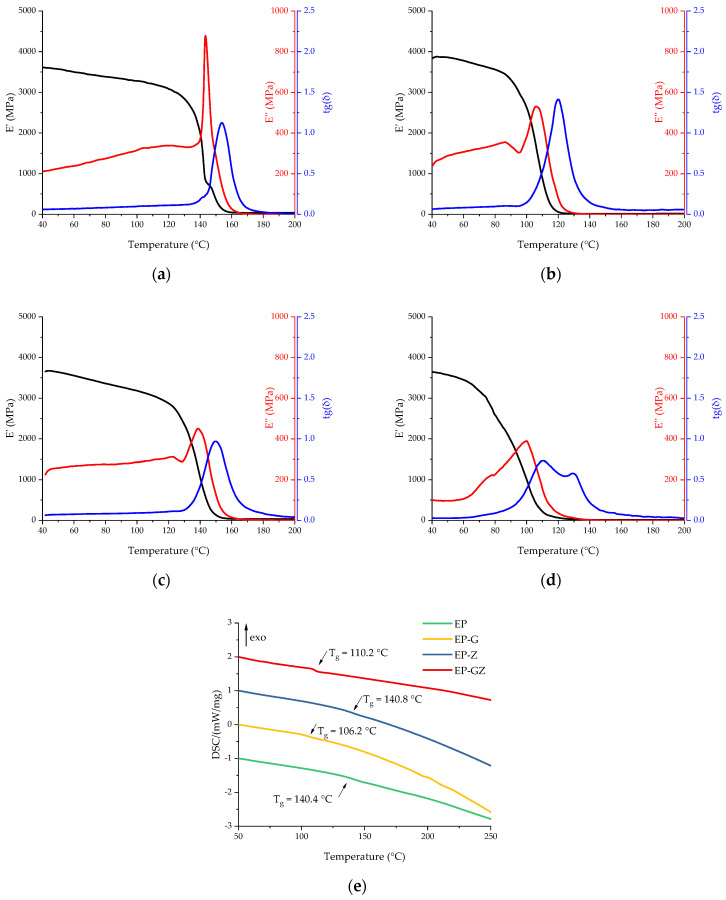
DMA curves of cured compositions: (**a**) EP, (**b**) EP-G, (**c**) EP-Z, and (**d**) EP-GZ. (**e**) Comparison of DSC curves of cured compositions obtained in the scanning mode.

All the compositions under study were cured according to the i-MTHPA technical datasheet. The curing program was 100 °C—1 h, 120 °C—2 h, and 150 °C—7 h, with the heating rate having isothermal steps of 2 °C/min. Thermal analysis of the cured compositions was carried out using DSC and DMA methods to determine the glass transition temperature. The resulting DSC and DMA curves are shown in Figure 8. The characteristic temperatures of the thermal effects are presented in Table 5.

The glass transition temperature of the cured initial composition was 140.4 °C according to the DSC data. The introduction of glycerol led to a drop in the glass transition temperature to 106.2 °C, which is associated with the introduction of a flexible aliphatic fragment into the polymer structure and a reduction of crosslink density. The introduction of zinc acetylacetonate did not have a significant effect on the glass transition temperature of the cured matrix, indicating that it did not integrate into the polymer network. A sample containing both glycerol and zinc acetylacetonate demonstrated a glass transition point of 110.2 °C according to the DSC data, which is slightly higher than for EP-G due to the Zn^2+^ complex formation. The influence of the introduced additives on the T_g_ obtained by the DMA method was the same. It is important to mention that the glass transition region became much wider in the case of formulations EP-G and EP-GZ, which could be caused by the formation of a non-uniform network.

The mechanical properties of the cured compounds under tension are presented in Table 6. The introduction of both glycerol and zinc acetylacetonate had a significant impact on the mechanical characteristics of the cured binders. According to the results obtained, with the introduction of additives, the elongation at rupture of the samples decreases significantly, and as a result, the tensile strength decreases significantly. Apparently, the introduction of additives causes embrittlement of the epoxy polymer matrix. The simultaneous introduction of glycerol and zinc acetylacetonate into the epoxy system leads to a drop in strength to 25.4 MPa, which corresponds to a drop of 67.5% relative to the base composition.

### 3.3. Reprocessibility of Resin Compositions

#### 3.3.1. Solubility of Cured Resin Compositions

The resulting cured compositions were tested for chemical resistance to a number of common solvents. All cured compositions showed a lack of solubility in acetone, dichloromethane, tetrahydrofuran, dimethyl sulfoxide, toluene, propylene glycol, EG, and water.

**Figure 9:**
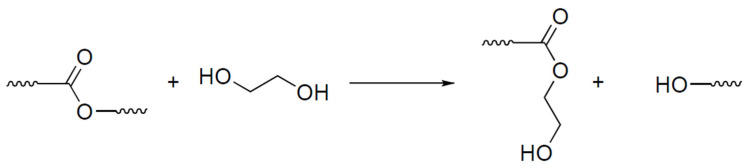
The proposed mechanism of the polymer matrix dissolution of ethyleneglycole.

For recycling purposes, the EG:NMP mixture with a ratio of nine to one by volume was selected. Ethylene glycol promotes the cleavage of the polymer matrix by the mechanism of transesterification, which is presented in Figure 9, while N-methylpyrrolidone provides better swelling of the polymer matrix, as evidenced in a recent publication [68].

Despite the insolubility of polymer matrices at room temperature, dissolution in the EG:NMP (9:1) system during boiling was carried out for their subsequent recycling. The dissolution times of polymer matrices were as follows: EP—35 h, EP-G—16 h, EP-Z—10 h, EP-GZ—5 h. The introduction of the Zn(Acac)_2_ as a transesterification catalyst led to a significant decrease in the dissolution time. However, even the introduction of the glycerol led to a significant decrease in dissolution time, which could have been caused by the reduced crosslinking degree of the resulting polymer. By introducing two modifiers at the same time, it was possible to reduce the dissolution time of the polymer matrix by seven times compared to the base composition caused by their synergistic effect, leading to a significant reduction in the dissolution time of the polymer matrix.

#### 3.3.2. Secondary Use of Dissolved Cured Resin Compositions

The dissolved polymer matrices were isolated from an aqueous suspension by extraction followed by solvent evaporation. The dried polymers were a sticky brown rubber-like mass. The DSC analysis showed the absence of any thermal effects in the range from 50 °C to 350 °C. The glass transition temperatures of dried secondary polymers determined by the DSC method are shown in Table 7. The difference between glass transition points might be caused by the residual solvent content after the evaporation.

**Figure 10:**
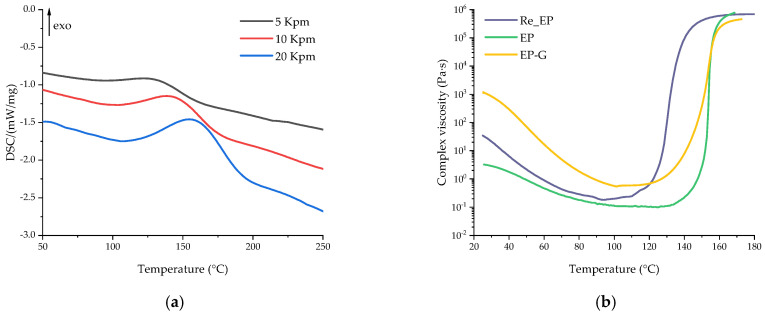
DSC curves of the uncured composition containing recycled polymers at different heating rates (**a**); complex viscosity dependence on temperature of the uncured composition containing recycled polymers, base composition, and the glycerol-containing composition (**b**).

For the secondary use of recycled polymer compositions, mixtures based on the basic composition containing 10 wt. % of secondary polymers were studied. As was observed by the DSC data, all the obtained recycled epoxy had an identical influence on the curing process of compositions and its thermal behavior. This could be explained by the removal of the zinc acetylacetonate during rinsing recycled epoxies with water. In this regard, further research was conducted only for a composition containing recycled EP. The key points of DSC scans are presented in Table 8. The DSC curves and complex viscosity dependence on temperature for a mixture with recycled composition EP are shown in Figure 10.

According to the DSC data, the addition of recycled epoxy resin had a significant effect on the curing process of the resin composition. Chemically recycled epoxy is characterized by a high content of hydroxyl groups formed during the dissolution of the polymer matrix (the proposed mechanism is shown in Figure 9). The presence of free hydroxyl groups catalyzes the process of opening the anhydride cycle, and thereby catalyzes the curing process, resulting in a decrease in onset temperature. The significant decrease in reaction enthalpy caused by the ring-opening reaction of anhydrides by free –OH groups indicates the catalytic efficiency of introduced epoxy recyclate. This also explains the significant increase in viscosity at room temperature due to the formation of branched molecules during the reaction of the anhydride hardener and –OH groups of recycled epoxy. Besides that, the gelation temperature of Re_EP was only 138 °C, which could be explained by the very high catalytic activity of recycled epoxy.

**Figure 11:**
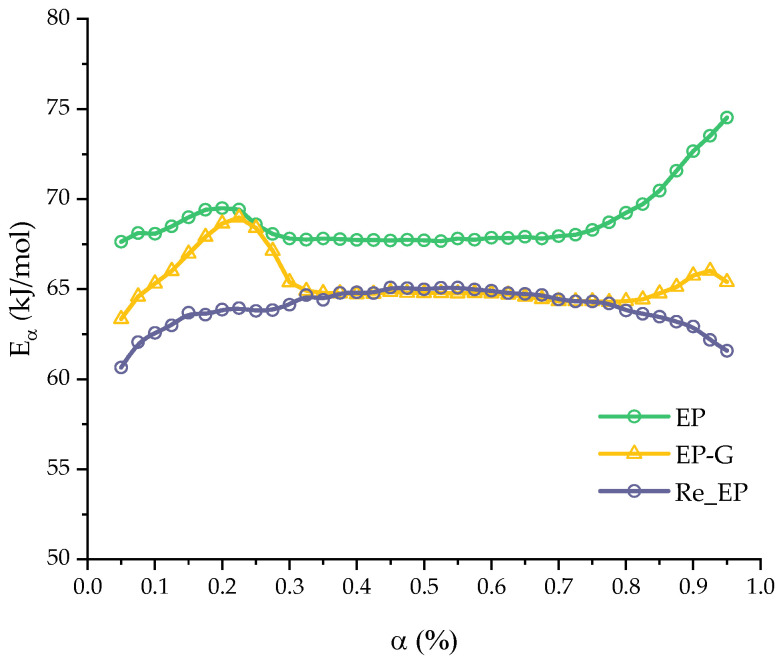
Activation energy $E_{\alpha }$ of the curing as a function of the extent of conversion α computed for EP-G and Re_EP compositions.

The activation energy curve for the Re_EP system was also calculated. The curve is shown in the Figure 11 in comparison with the curves for systems EP and EP-G systems. Average value of activation energy for the system Re_EP is given in Table 9.

**Figure 12:**
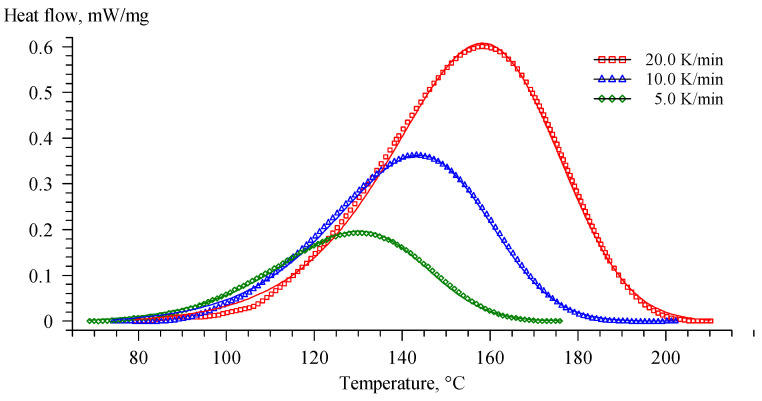
Comparison of the experimental DSC curves (symbols) with those estimated (solid lines) using Cnm model for Re-EP.

The system with the introduction of a secondary vitrimer behaved like the EP-G system during curing (Figure 12). The absence of the pre-exponential factor $A_{1}$ (Table 10), the similarity of the reaction orders n and m, and, what is important, the activation energies indicate the similarity of the processes occurring both in the system with the introduction of glycerol and in systems with a recycled vitrimer. This behaviour is also confirmed by isoconversional analysis data.

**Figure 13:**
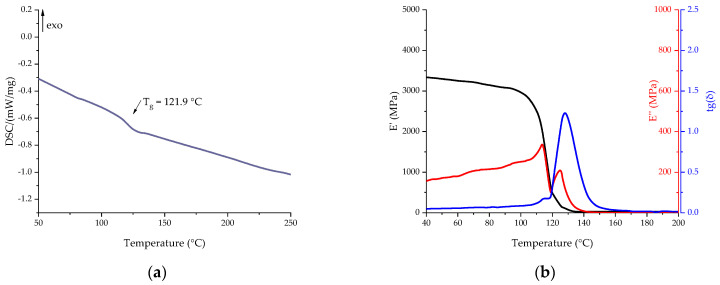
DSC (**a**) and DMA (**b**) curves of cured Re_EP composition in the scanning mode.

Compositions with secondary vitrimers were cured according to the same curing program as primary compositions. Thermal analysis of the cured binders obtained was carried out using DSC and DMA methods to determine the glass transition temperature. The results of the DSC and DMA studies are shown in Figure 13. The key characteristics of the measurements are presented in Table 11.

The thermal properties of the cured composition containing recycled EP turned out to be lower than the base composition. According to the DSC data, the glass transition temperature was 121.9 °C. The presence of a high content of hydroxyl groups formed during the dissolution of the polymer matrix, as well as flexible aliphatic fragments of ethylene glycol, leads to a decrease in the glass transition temperature. However, the drop in glass transition temperature, both according to the DMA data and according to the DSC data, with the addition of a recycled epoxy is lower than with the addition of glycerol. The temperature dependences of the elastic modulus for the obtained compositions with various secondary polymers do not differ significantly from each other, which confirms the hypothesis of the identity of the recycled epoxy vitrimers.

The introduction of recycled vitrimer resin into the base composition had a significant impact on the mechanical characteristics of the resin. The elongation at rupture dropped to 1.00 ± 0.14%, and as a result, the tensile strength dropped to 29.6 ± 3.7 MPa (a 62% drop relative to the base composition). Apparently, the introduction of recycled vitrimer resin causes embrittlement of the epoxy polymer matrix. Meanwhile, the modulus of elasticity remained at the same level as the base composition (3.02 ± 0.08 GPa for recycled-containing resin versus 3.03 ± 0.13 GPa for EP).

Such a significant decrease in elongation at rupture and mechanical strength requires further tuning of the presented formulation. However, the modulus of elasticity remained on the same level, and the glass transition point decreased only by 20 °C, which is less than in the case of the use of glycerol. Besides that, the introduced recycled epoxy showed higher catalytic efficiency than glycerol, which could be used to decrease curing time and the maximum curing temperature.

## 4. Conclusions

In this study, we report on the investigation of vitrimers based on epoxy-anhydride resin. When the epoxy resin is cured with anhydride hardeners, a polyester structure is formed, which can undergo reversible dynamic transesterification reactions, as a result of which, a topological restructuring of the polymer network is possible. However, improving recyclability requires the introduction of modifiers to accelerate transesterification reactions. During this study, the effect of glycerol and zinc acetylacetonate on the kinetics of curing epoxy vitrimers, as well as the process of chemical recycling, and technological and operational properties of the obtained vitrimers were studied.

Using the isoconversional method, it was shown that the activation energy of the curing processes of all primary compositions is almost independent of the extent of conversion in a quite wide range. Based on these data, average activation energy values were obtained.

The introduction of glycerol significantly accelerates the curing process, reducing the mean value of isoconversional activation energy from 69 kJ/mol to 65 kJ/mol. The introduction of zinc acetylacetonate increases both the polymerization temperature and activation energy (from 69 kJ/mol to 146 kJ/mol). Simultaneous introduction of both components shows a decrease in the polymerization temperature relative to the composition containing only zinc acetylacetonate and also reduces the activation energy to a value of ~141 kJ/mol. Apparently, with the introduction of zinc acetylacetonate, the formation of complexes occurs, which impedes the polymerization process of the resin.

It was shown that a kinetic model consisting of parallel reactions with single activation energy but with different values of other parameters describes kinetic data well. The obtained activation energy values are comparable with the average values obtained by the isoconversional method. This proves the consistency of the calculated activation energies.

Thermomechanical and mechanical properties of binders were also studied. The introduction of glycerol decreases the glass transition temperature of the cured matrix, while zinc acetylacetonate has practically no effect on T_g_. However, the use of modifiers significantly reduces the mechanical tensile strength, which is probably due to embrittlement of the polymer matrix, since the ultimate deformation for a composition with two additives introduced drops by more than 80% relative to the base composition.

The use of selected modifiers significantly affects the chemical dissolution of the cured vitrimers by the transesterification reaction between the polyester structure of the cured formulation and EG molecules. The base epoxy composition was dissolved during reflux in the EG/NMP system for 35 h. The introduction of glycerol in the amount of 10 phr to base resin composition reduced the dissolution time to 16 h (reduction by 54%); the introduction of zinc acetylacetonate in the amount of 6.73 phr to base resin composition reduced it to 10 h (reduction by 71%). The simultaneous use of two modifiers in the above-mentioned amounts reduced the dissolution time to 5 h (reduction of 86%).

The possibility of the secondary use of dissolved polymers as epoxy binder modifiers was considered. The influence of recirculate addition on the curing process and the properties of the epoxy-anhydride resin were studied. Recycled polymer showed high catalytic activity towards curing epoxy-anhydride resin, which could be useful for lowering the maximum curing temperature, curing time, and activation energy (~64 kJ/mol). A high concentration of hydroxyl groups of the dissolved polymer probably leads to an increase in the initial viscosity. At the same time, the use of a recycled vitrimer reduces the glass transition temperature of the cured matrix to 122 °C due to flexible ethylene glycol fragments in the structure of the dissolved polymer. Meanwhile, the introduction of 10 w.t. % of recycled epoxy vitrimer to the base composition led to the drop of tensile strength to 29.6 ± 3.7 MPa (a 62% drop relative to the base composition), which is associated with a decrease in the ultimate deformation. Observed performance of the studied formulation could be helpful for a better understanding of the influence of recirculate addition on properties and further applications of the epoxy-anhydride resins.

## Figures and Tables

**Table 1:** Formulations of the mixtures under study in mass parts.

Component	EP	EP-G	EP-Z	EP-GZ
DER 330	100	100	100	100
i-MTHPA	85	85	85	85
Glycerol	-	10	-	10
Zn(AcAc)_2_	-	-	6.73	6.73
4-MIm	1.85	1.85	1.85	1.85

**Table 2:** Characteristic temperatures of DSC curves of uncured samples.

Formulation Index	Heating Rate, °C/min	T_onset_, °C	T_peak_, °C	T_end_, °C	ΔH, J/g
EP	5	108.2	129.6	146.7	326.4
	10	122.3	143.5	160.9	347.2
	20	129.1	156.0	177.2	349.0
EP-G	5	99.9	123.4	152.5	229.0
	10	100.6	135.5	169.1	235.3
	20	112.6	149.9	184.3	249.2
EP-Z	5	127.9	146.1	161.8	336.2
	10	142.8	160.6	176.8	348.9
	20	149.7	172.3	192.1	350.4
EP-GZ	5	120.3	141.4	161.3	244.9
	10	127.8	152.8	175.4	249.5
	20	141.3	167.5	193.9	272.4

**Table 3:** The values of the mean activation energy $E_{\alpha }$ and the corresponding standard error.

Formulation Index	$E_{\alpha }$ and Standard Error, kJ/mol
EP	68.79 ± 0.28 *
EP-G	65.32 ± 0.22 *
EP-Z	75.61 ± 0.17 **
EP-GZ	72.36 ± 0.27 *

*—average value of $E_{\alpha }$ at α ranging from 0.05 to 0.95, **—average value of $E_{\alpha }$ at α ranging from 0.15 to 0.70.

**Table 4:** The values of the kinetic parameters obtained for the Cnm model.

Formulation Index	$\log\;A_{1}$, log 1/s	$\log\;A_{2}$, log 1/s	E, kJ/mol	n	m	B	R^2^
EP	6.59	7.53	71.1	1.62	1.65	8.590	0.999
EP-G	-	6.42	65.2	1.20	0.35	-	0.999
EP-Z	6.95	8.05	78.5	1.54	1.30	12.417	0.997
EP-GZ	6.26	7.25	72.4	1.64	1.01	9.772	0.999

**Table 5:** Characteristic temperatures of DSC and DMA curves of cured samples.

DSC			DMA	
Formulation Index	T_g_ Middle, °C	E′ Onset, °C	E″ Peak, °C	Tan (δ) Peak, °C
EP	140.4	141.9	142.7	152.9
EP-G	106.2	106.2	106.1	120.6
EP-Z	140.8	136.2	138.3	149.1
EP-GZ	110.2	75.6	99.6	110.2

**Table 6:** Results of tensile tests of cured resins.

Composition	σ, MPa	E, GPa	δ, %
EP	78.3 ± 5.9	3.03 ± 0.13	4.40 ± 0.95
EP-G	58.8 ± 4.6	3.12 ± 0.04	2.20 ± 0.22
EP-Z	53.9 ± 13.4	3.09 ± 0.06	2.09 ± 0.64
EP-GZ	25.4 ± 5.5	3.17 ± 0.08	0.81 ± 0.17

**Table 7:** Glass transition points of secondary resin compositions.

Formulation Index	T_g_ Middle, °C
EP	9.1
EP-G	34.9
EP-Z	6.1
EP-GZ	−4.6

**Table 8:** Characteristic temperatures of DSC curves of uncured Re_EP.

Formulation Index	Heating Rate, °C/min	T_onset_, °C	T_peak_, °C	T_end_, °C	ΔH, J/g
Re_EP	5	94.0	131.3	161.1	80.8
	10	106.1	143.4	173.0	85.8
	20	114.5	158.5	192.2	88.0

**Table 9:** The values of the mean activation energy $E_{\alpha }$ and the corresponding standard error for Re_EP system in comparison with EP and EP-G systems.

Formulation Index	$E_{\alpha }$ and Standard Error, kJ/mol
EP	68.79 ± 0.28 *
EP-G	65.32 ± 0.22 *
Re_EP	63.95 ± 0.18 *

*—average value of $E_{\alpha }$ at α ranging from 0.05 to 0.95.

**Table 10:** The values of the kinetic parameters obtained for the Cnm model for Re_EP system.

Formulation Index	${\log\;A}_{1}$, log 1/s	${\log\;A}_{2}$, log 1/s	E, kJ/mol	n	m	B	R^2^
Re_EP	-	6.07	64.3	1.05	0.25	-	0.999

**Table 11:** Characteristic temperatures of DSC and DMA curves of cured Re_EP sample.

Formulation Index	DSC	DMA		
	T_g_ Middle, °C	E′ Onset, °C	E″ Peak, °C	Tan (δ) Peak, °C
Re_EP	121.9	110.2	114.3	128.6

## Data Availability

The data presented in this study are available on request from the corresponding author.
